# Early-Life Sublethal Exposure to Thiacloprid Alters Adult Honeybee Gut Microbiota

**DOI:** 10.3390/genes15081001

**Published:** 2024-07-31

**Authors:** Bin Li, Xiasang Chen, Li Ke, Pingli Dai, Yuan Ge, Yong-Jun Liu

**Affiliations:** 1 State Key Laboratory of Resource Insects, Institute of Apicultural Research, Chinese Academy of Agricultural Sciences, Beijing 100193, Chinajnklst@163.com (L.K.);; 2 State Key Laboratory of Urban and Regional Ecology, Research Center for Eco-Environmental Sciences, Chinese Academy of Sciences, Beijing 100085, China

**Keywords:** thiacloprid, larval stage, microbiome, 16S rDNA sequencing

## Abstract

Thiacloprid, a neonicotinoid pesticide, is known to affect the gut microbiome of honeybees, yet studies often focus on immediate alternations during exposure, overlooking long-term microbiological impacts post-exposure. This study investigates the influences of sublethal thiacloprid administered during the larval developmental stage of honeybees on physiological changes and gut microbiota of adult honeybees. We found that thiacloprid exposure increased mortality and sugar intake in emerged honeybees. Using 16S rDNA sequencing, we analyzed intestinal microbial diversity of honeybees at one and six days post-emergence. Our findings reveal a significant but transient disruption in gut microbiota on day 1, with recovery from dysbiosis by day 6. This study emphasizes the importance of evaluating chronic sublethal exposure risks of thiacloprid to protect honeybee health.

## 1. Introduction

Honeybees are primary pollinators that play important roles in global ecosystems and agroecological biodiversity [1]. Honeybees are vulnerable to toxins as they forage for pollen and nectar [2]. Neonicotinoids, which are used in agricultural processes worldwide due to their high efficiency and low toxicity to vertebrates, can be toxic to non-target bees [3]. They are taken up systemically, allowing neonicotinoids to transfer from plant roots to environments relevant to bee-relevant matrices [4], elevating exposure risks for pollinators like honeybees.

It has been demonstrated that exposure to sublethal concentrations of neonicotinoid pesticides, such as imidacloprid, clothianidin, and thiamethoxam, can accumulate in pollen and nectar and cause chronic damage and pose substantial risks to bee colony development [5]. Negative effects of neonicotinoids have been demonstrated in previous studies, including inhibited growth [6], compromised immunity [7], increased disease susceptibility [8], and impaired queen fertility [9]. They also impact bee behaviors, like decreasing foraging success, navigation efficiency [10], and impaired cognitive and colony communication [11,12]. Thiacloprid was considered less toxic to bees than other neonicotinoids like imidacloprid or thiamethoxam [13,14], but it also targets neuronal nicotinic acetylcholine receptors [15] and could disrupt neural transmission [16], potentially leading to neural persistent excitation and paralysis.

The honeybee gut microbiota is composed of 8–10 core intestinal flora [17] and is established shortly after emergence [18]. Honeybee gut microbiota play important functions in digestion and absorption of nutrients [19], metabolism [20,21], and immunity [22]. A stable gut microbiota is essential for honeybee health [23]. Neonicotinoids, including thiacloprid, have been shown to disrupt microbiota in adults [24,25] and larval bees [26]. Our preliminary research suggests that thiacloprid exposure among adult honeybees resulted in dysbiosis of their gut microbiota [27]. The composition of the honeybee microbiota is also related to the age of bees [28]. Honeybees begin acquiring their gut microbiota within hours of emerging from the pupa, and full colonization usually occurs within six days [18]. However, the impacts of prolonged thiacloprid exposure during the larval stage on the gut microbial community of adult honeybees remain unclear.

This study investigates whether sublethal concentrations of thiacloprid, administered during the larval phase, alter the gut microbiota of emerged honeybees on day 1, when initial colonization begins, and on day 6, when the gut microbiota is fully established. Under laboratory conditions, honeybee larvae were exposed to thiacloprid, and their survival rates, sucrose intake, and body weight were recorded for 6 days after the honeybee emerged. Furthermore, the impacts of early larvae exposure to thiacloprid on adult honeybee gut microbiota communities were assessed. This research sheds light on the long-lasting effects of sublethal thiacloprid on honeybee development and the early stages of gut microbiota construction.

## 2. Materials and Methods

### 2.1. Chemicals and Reagents

Thiacloprid (Sigma-Aldrich, St. Louis, MO, USA) was dissolved in DMSO (dimethyl sulfoxide), and then diluted with freshly prepared food (the formula is described in detail in [29]) to the required concentrations of 0.5 mg/L and 1.0 mg/L. The thiacloprid concentrations are within the range of thiacloprid found in the field [7,30]. DMSO concentrations are below 0.001%; the impacts of DMSO at such a low level on honeybees are negligible [31].

### 2.2. Sample Collection

All honeybees were collected from four colonies at the Institute of Apicultural Research apiary (39°59′ N, 116°11′ E), Chinese Academy of Agricultural Science (Beijing, China). The colonies did not show signs of mite infestation or common viral infections, such as Israeli acute paralysis virus, black queen cell virus, or deformed wing virus). Honeybee queens oviposited on blank brood frames; on the 3rd day after the queen bee spawned, the larvae were transferred to sterile 48-well plates and reared at 35 °C and 90% relative humidity in darkness in an incubator (RXZ—380C, Ningbo, China). Each treatment group (0 mg/L, 0.5 mg/L, and 10 mg/L) contained four replicates of 96 honeybee larvae from each colony. According to the in vitro rearing protocols outlined by Schmehl et al. [29], freshly prepared food containing different dosages of thiacloprid (0.0 mg/L, 0.5 mg/L, and 1.0 mg/L) was administrated throughout the larval stage (6 days). The diet was replenished daily. Upon transitioning to pupae on the 7th day, they were transferred to 24-well plates and incubated at 35 °C with 75% relative humidity. After emergence, the emerged adult bees were housed at 30 °C and 50% relative humidity for an additional 6 days. To prevent contamination with new intestinal bacteria from pollen, honeybees were fed 50% sterile sucrose solution. Honeybee survival rate, sucrose intake, and body weight were measured for 6 days after emergence. For microbial sequencing, 10 honeybees were collected randomly from different replicates of each treatment group, and their intestines were excised for further DNA extraction. Thiacloprid residues in honeybee abdomens were measured using LC-MS/MS. Each group had 3 biological replicates, with 5 bee samples mixed in each replicate.

### 2.3. DNA Extraction and Sequencing

The honeybee intestinal DNA was extracted using a Fast DNA SPIN Kit (MP Biomedicals, Santa Ana, CA, USA). Purified DNA was evaluated for quality using a NanoDrop 2000 spectrophotometer (ThermoFisher, Shanghai, China). Only high-purity samples were selected for subsequent sequencing. The V3–V4 region of the bacterial 16S rRNA gene was amplified using the PCR with primer set 338 F (5′-ACT CCTACGGGAGGCAGCAG-3′) and 806 R (5′-GGACTACHVGGGTWTCTA-AT-3′). Primers were incorporated with 6-bp oligonucleotide barcodes for sample identification. The purified PCR products were quantified using the QuantiFluor™-ST DNA System (Promega, Madison, WI, USA) and sequenced on an Illumina MiSeq platform (Illumina, Santiago, CA, USA).

### 2.4. Data Analyses

Illumina sequences were processed using Quantitative Insights Into Microbial Ecology (QIIME™, an open-source project primarily developed at Knight Lab at the University of Colorado Boulder, Boulder, CO, USA). FASTQ sequencing files were initially fileted as described in Liu et al. [27]. High-quality data sets were combined utilizing FLASH 1.2.11 (https://ccb.jhu.edu/software/FLASH/index.shtml, accessed on 29 June 2024). Subsequently, operational classification units (OTUs) were grouped together using UPARSE 7.0.1090 (http://www.drive5.com/uparse/, accessed on 29 June 2024) with a threshold of 97% similarity, and any chimeric sequences were identified and eliminated with UCHIME. Taxonomic assignments were made for these OTUs against the SILVA 138 database (http://www.arb-silva.de, accessed on 29 June 2024), and a classification analysis was conducted using the RDP classifier 2.11 (https://sourceforge.net/projects/rdp-classifier/, accessed on 29 June 2024) with a predefined threshold of 0.7. Finally, OTUs with an abundance of less than 0.1% were removed to eliminate pyrosequencing errors. All raw sequencing data have been deposited in the NCBI database (Bioproject ID: PRJNA940397).

Data analyses were performed on the Majorbio cloud platform (https://cloud.majorbio.com, accessed on 29 June 2024). Alpha diversity was represented by Chao1, ACE, phylogenetic diversity (pd), and the Shannon index. Beta diversity was evaluated using Jaccard distance and non-metric multidimensional scaling analysis (NMDS). Additionally, to explore the relationship between microbial community composition and environmental conditions, distance-based redundancy analysis (db-RDA) was performed. The niche width index was calculated to assess the extensiveness and homogeneity within microbial communities, which is implemented using spaa packages in R v4.4.1. To discern the influence of deterministic versus stochastic processes on community formations, we computed the modified stochasticity ratio (MST) using the NST package in R [32]. MST can better quantitively measure the stochasticity in assembly [33,34], and its values over 50% indicate stochastic dominance, whereas values less than 50% indicate the prevalence of deterministic processes.

### 2.5. Statistics

Honeybee survival rates were evaluated with the log-rank paired test, with Bonferroni-adjusted *p*-values. Sucrose intake, body weight, as well as thiacloprid residue were compared using the Kruskal–Wallis test followed by Dunn’s multiple comparison test. To assess the statistical significance of NMDS, analyses of similarities (ANOSIM) with 999 permutations were employed. Permutest analysis was used to determine the significance of db-RDA results. The Kruskal–Wallis test followed by Dunn’s multiple comparison test was used to compare alpha diversity indices, beta diversity, MST values, and niche width indices across thiacloprid treatment groups.

Statistical analyses were carried out using R (http://www.r-project.org/, accessed on 29 June 2024), SPSS v20.0.0.0, or GraphPad Prism v8.3.0.

## 3. Results

### 3.1. Thiacloprid Larval Exposure Affects the Physical Status of Adult Honeybees

**Figure 1:**
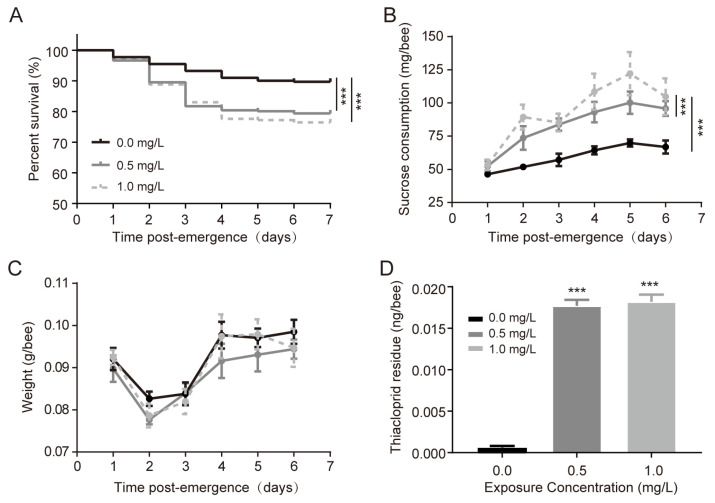
Impact of thiacloprid larvae exposure on the survival rate (**A**), sucrose intake (**B**), and body weight (**C**) of emerged adult honeybees over time. Thiacloprid residue of newly emerged bees within each treatment group (**D**). The sample size for each group is 4; all results are shown as the mean ± SE. *** *p* < 0.001.

An assessment of various physiological aspects in adult honeybees, including survival, sugar intake, and body weight, were measured for six days after emergence. The survival rates of honeybees significantly decreased in the thiacloprid treatment groups compared to the control group (*p* < 0.001), as shown in Figure 1A, with a 10% and 13% decrease at 0.5 and 1.0 mg/L concentrations, respectively. Larvae exposed to thiacloprid exhibited significantly increased sucrose intake in adult honeybees in thiacloprid-treated groups (*p* < 0.001 in 1.0 mg/L groups), as illustrated in Figure 1B. However, honeybee body weight disclosed no significant difference between thiacloprid treatment groups and the control group (0.5 mg/L vs. control, *p* = 0.127; 1.0 mg/L vs. control, *p* = 0.514), as depicted in Figure 1C. Analysis of thiacloprid residues in honeybee abdomens showed substantially higher levels in the thiacloprid treatment groups compared with the control (Figure 1D, *p* < 0.001), with no statistical differences between thiacloprid treatment groups of honeybees.

### 3.2. Thiacloprid Modulates the Diversity of Adult Honeybee Gut Microbiota

**Figure 2:**
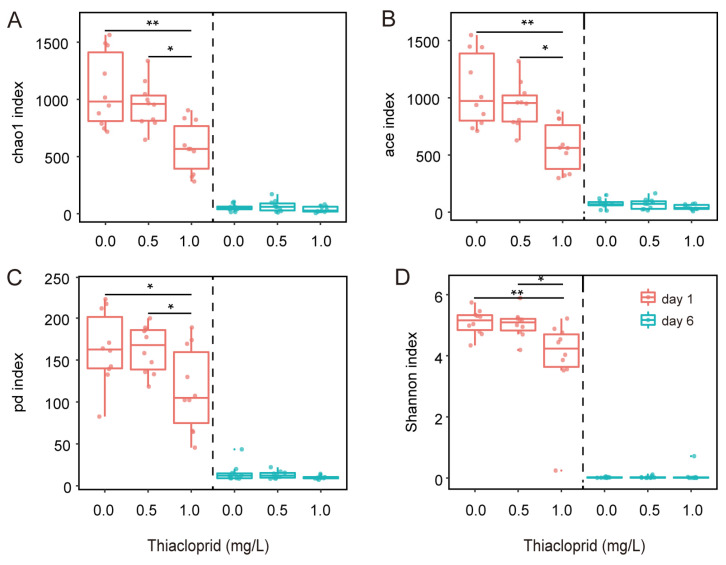
Alpha diversity of the gut microbiome in honeybees exposed to thiacloprid and control bees. (**A**) Chao1 and (**B**) ACE indices reflecting OTU abundance, and (**C**) pd index and (**D**) Shannon index depicting OTU diversity in all samples (mean ± SE, n = 10 in each group). Statistical analysis was performed using the Kruskal–Wallis test, followed by Dunn’s multiple comparison. * *p* < 0.05, ** *p* < 0.01.

To assess species diversity within the microbiota community, alpha diversity including Chao1, ACE, phylogenetic diversity, and the Shannon index were calculated on day 1 and day 6 (Figure 2). The Kruskal–Wallis test was performed to compare the alpha diversity of honeybee gut microbiota in different thiacloprid treatment concentrations. The results indicated that on day 1 the alpha diversity of honeybee gut microbiota significantly decreased in the thiacloprid treatments groups compared to the control group (Figure 2A–D, 1.0 mg/L vs. control, *p* < 0.01 for Chao1, *p* < 0.01 for ACE, *p* < 0.05 for pd, *p* < 0.01 for Shannon index). By day 6, the alpha diversity of honeybee gut microbiota was no longer significantly different from the control group (*p* > 0.05 for all indices).

**Figure 3:**
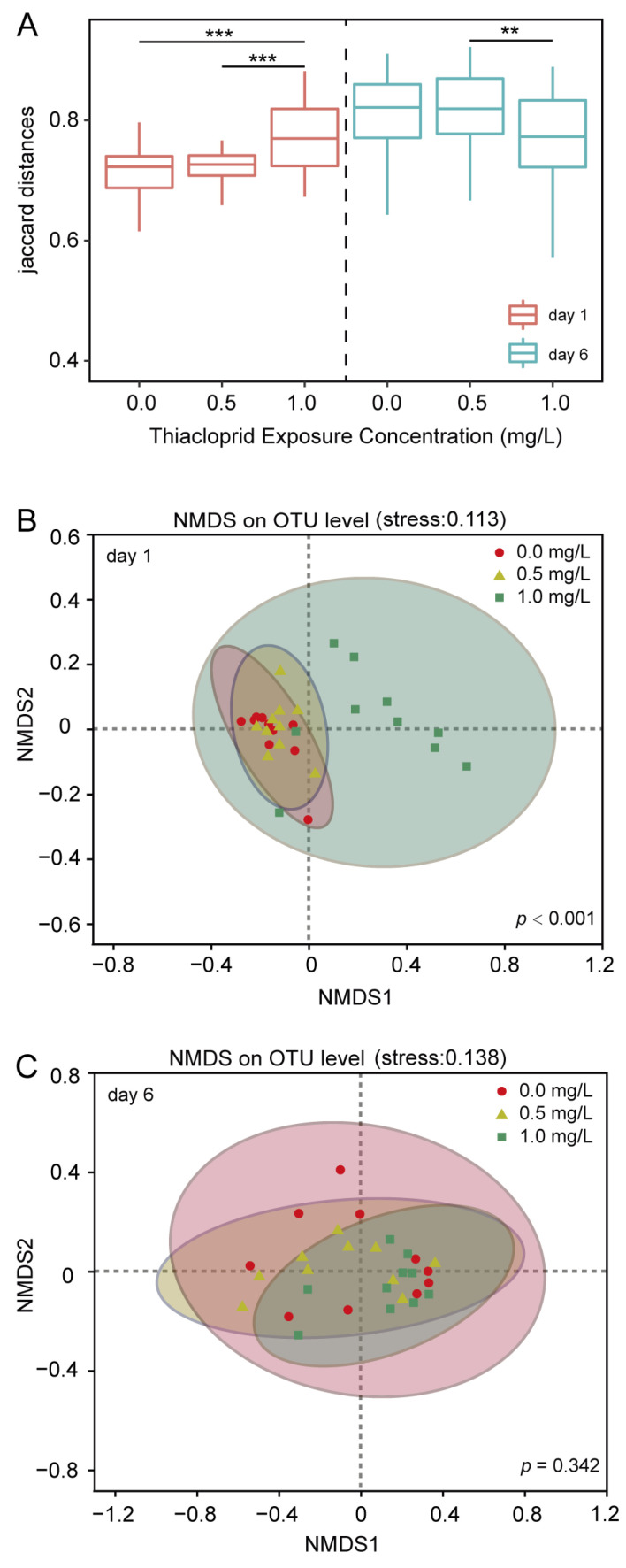
Beta diversity of the microbiome within each thiacloprid treatment group. (**A**) box-and-whisker plot illustrating beta diversity based on Jaccard distance in control and thiacloprid-exposed groups. Statistical analysis was performed using the Kruskal–Wallis test, followed by Dunn’s multiple comparison. Data are presented as mean ± SE, ** *p* < 0.01, *** *p* < 0.001. (**B**) NMDS (non-metric multidimensional scaling) analysis of intestinal microbial communities in each treatment group on day 1 (ANOSIM, *p* < 0.001) and (**C**) on day 6 (ANOSIM, *p* = 0.342).

Analysis of beta diversity in the honeybee microbiome using Jaccard distances indicated that community dissimilarity increased in the higher thiacloprid treatment group on day 1 (1.0 mg/L vs. control, *p* < 0.001, Figure 3A). However, by day 6, Jaccard distances decreased significantly with increasing thiacloprid concentrations (0.5 mg/L vs. 1.0 mg/L, *p* < 0.01). The honeybee gut microbial community diversity across different thiacloprid treatment groups was also compared using NMDS as shown in Figure 3B,C. There were notable disparities in the gut microbial community composition between control and thiacloprid treatment groups on day 1 (Figure 3B; ANOSIM with 999 permutations, *p* < 0.001), but not on day 6. The community of microbes was found to be more concentrated in the control group, while thiacloprid exposure led to a marked divergence, particularly at higher concentrations. In addition, on day 6, the microbial community compositions from two thiacloprid treatment groups converged, with no significant differences detected (Figure 3C; ANOSIM with 999 permutations, *p* = 0.342), suggesting a transient impact of thiacloprid on gut microbial diversity on day 1, but lasting to day 6.

### 3.3. Factors Influencing the Early Establishment of Gut Microbiota in Adult Honeybees

**Figure 4:**
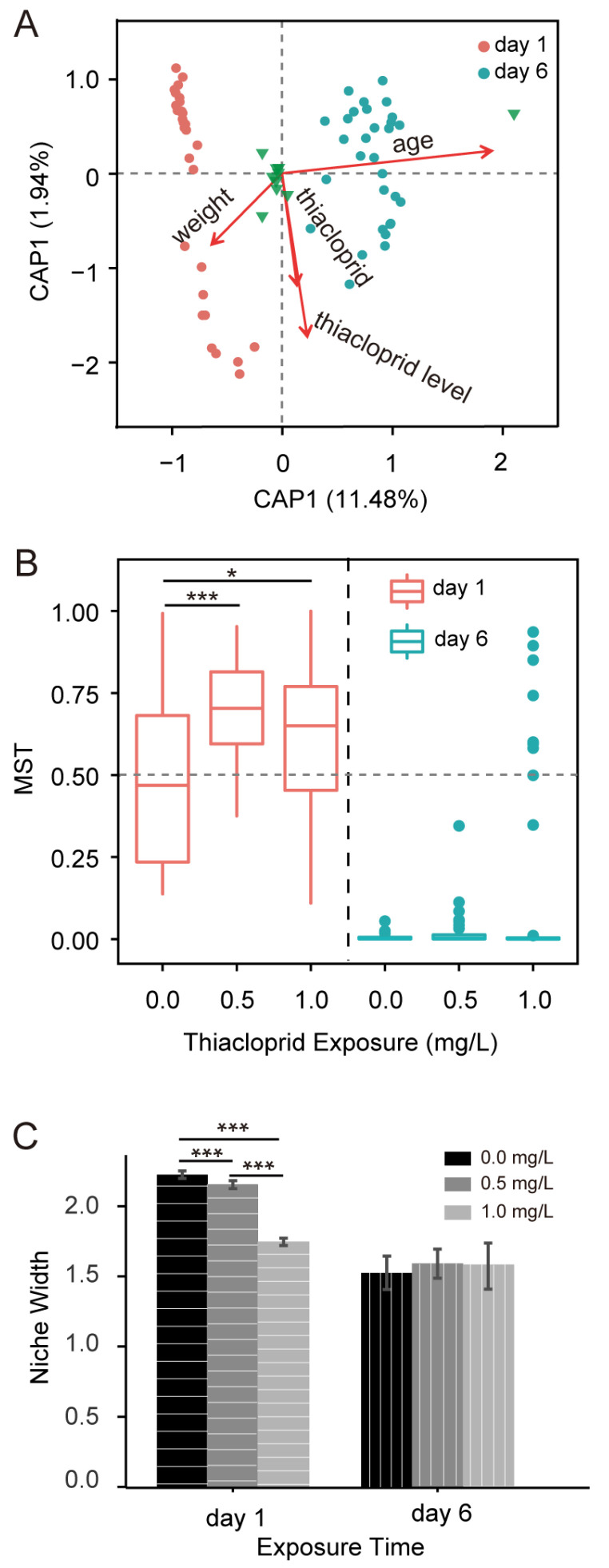
Factors affecting honeybee gut microbial community construction. (**A**) db-RDA analysis reveals the gut bacterial communities in response to environmental factors. Dots of varying colors or shapes represent different sampling days (day 1 and day 6), with green triangles denoting microbial species at the OTU level. The strength and direction of the red arrows indicate relative correlations. (**B**) MST values for gut microbial communities in different treatment groups on day 1 and day 6. (**C**) Ecological niche width of intestinal microbial communities in honeybees on day 1 and day 6 under different concentrations of thiacloprid. Statistical analysis was performed using the Kruskal–Wallis test, followed by Dunn’s multiple comparison. Data are presented as mean ± SE, * *p* < 0.05, *** *p* < 0.001.

The honeybee gut microbiota is influenced by environmental factors. To examine the relationship between environmental factors and the gut microbial community, distance-based redundancy analysis (db-RDA) was calculated. Figure 4A illustrates that the gut microbial community was significantly influenced by bee age and thiacloprid exposure. Moreover, temporal changes (day 1 to day 6) exerted a greater influence than thiacloprid concentration (refer to Table 1 for details).

In order to assess the extensiveness and homogeneity of microbial distribution within a community, niche width analysis was performed. Niche width analysis underscored the age-related shifts within the gut microbiota: honeybees exhibited a wider niche index on day 1, which narrowed by day 6 (Figure 4C), particularly under thiacloprid exposure. The temporal impact was profound, with initial variations in niche width correlating with thiacloprid doses on day 1 and a significant decrease found in the 0.5 and 1.0 mg/L thiacloprid treatments in comparison with the control group (0.5 or 1.0 mg/L vs. control, *p* < 0.001). Yet, these differences were not sustained by day 6. The results suggest thiacloprid treatment significantly reduced the homogeneity of the microbiota of honeybees on day 1, whereas it had less effect on day 6.

To examine the assembly mechanisms of the gut microbiota, we further assessed the interplay between stochastic and deterministic building procedures in honeybee gut microbiota formation across treatment groups using the modified stochasticity ratio, as shown in Figure 4B. On day 1, a shift from deterministic to stochastic processes as thiacloprid concentration increased was observed, as MST values rose from 0.484 to 0.618 (Figure 4B; 0.5 mg/L vs. control, *p* < 0.001; 1.0 mg/L vs. control, *p* < 0.05), suggesting increased randomness in microbial community structure with increasing thiacloprid doses, likely a response to its residual toxicity. Nevertheless, by day 6, the stochastic influence of thiacloprid receded, with MST values indicating a reversion to deterministic community assembly processes, indicating a reduction in thiacloprid’s stochastic impact over time.

## 4. Discussion

Our findings demonstrate that when larval-stage honeybees are exposed to sublethal concentrations of thiacloprid, this results in changes in survival rate and sucrose intake in the subsequently emerged bees. In addition, we reveal that early-life thiacloprid exposures also change adult bees’ gut microbial communities. This finding extends current understanding, which has primarily focused on the immediate effects of neonicotinoids on the microbiota of honeybees [35,36], and bumble bees [37]. Our work bridges a critical gap by demonstrating that brief exposure to thiacloprid throughout the larval phase of honeybees can indeed exert long-lasting effects on the gut microbiota of adult honeybees post-emergence.

Consistent with our prior study, we found that thiacloprid exposure throughout the honeybee larval phase significantly influences the survival of emerged adult bees [6]. This reduction is potentially attributable to the negative effects on honeybee blood cells and cystic membranes [7]. Further studies support our findings, showing that neonicotinoids like thiacloprid adversely affect bee larvae and queens [5]. While field studies have reported negligible effects from chronic sublethal exposure to thiacloprid on bee colonies [38], the discrepancy with our laboratory findings highlights the influence of experimental conditions, such as field and laboratory settings, as well as that other factors like bee age and food might influence susceptibility to thiacloprid. Intriguingly, our results also reveal that thiacloprid-exposed bees increased their sugar-water consumption without a corresponding weight gain, suggesting a possible disruption of energy metabolism pathways. This hypothesis is supported by the observation that thiacloprid exposure modulates gene expression related to cellular functions and metabolic processes [6].

Antibiotics or pesticides disrupt honeybee microbiomes [7,39]; our study is in accordance with previous work and further provides novel insights into the temporal dynamics of these effects. We discovered thiacloprid treatment in the larval stage dramatically reduced gut microbial alpha diversity on the first day post-emergence, as evidenced by the richness of evaluation (Chao1 and ACE indices) and evenness estimates (pd and Shannon indices). However, this disruption in alpha diversity was transient, with no significant differences observed by the sixth day. The perturbation extends to the beta diversity of the bee microbiota, where we noted a rise in the Jaccard distance, indicative of a distinct microbial community structure on day 1; however, such alternations were not sustained until day 6. NMDS results also confirm similar results, which further corroborate the immediate but not lasting impact of thiacloprid on gut microbial composition. Our findings are consistent with the previous study that insecticides can perturb the honeybee gut microbiome [7,27,39,40], with potential downstream effects on host metabolism and immunity [22], activating antioxidant defenses and inducing epithelial cell damage [25,41], and thereby increasing the bees’ susceptibility to pathogenic bacteria. This phenomenon might explain the reduced honeybee survival rates caused by thiacloprid exposure in our experiment. Furthermore, neonicotinoids have been documented as potential providers of carbon and energy from some bacteria [42,43]. Different concentrations of neonicotinoid treatments may be related to alternations in specific microbial flora or microbiome genes responsible for maintaining metabolic function in the host gut, suggesting a possible interaction between thiacloprid, specific gut microbes, and the metabolic processes they govern.

The study revealed that thiacloprid use, along with bee age, plays an important role in the composition of the honeybee gut microbiome. Our findings are in line with earlier studies which highlight age-dependent characteristics in the core gut species [18,44] and point out the more stable nature of the gut microbiota in adult workers in contrast to larvae [45]. Our data also confirm that the impact of thiacloprid is less on more mature honeybees on day 6. The concentration of neonicotinoid in honeybees could decrease due to metabolism and excretion [46], reducing toxicity and allowing damaged microbial communities to gradually recover. Honeybees on day 6 have a more stable gut microbiome and enhanced immunity than newly emerged honeybees [18], enabling them to better resist the effects of thiacloprid on their gut microbiota [47]. Furthermore, bacterial species might experience seasonal fluctuations in the relative proportions of their core constituents [48], and these shifts are further influenced by geographic regions [49]. Environmental conditions like pesticide exposure [50] or diet type also play an important role in microbiome community [51].

Furthermore, thiacloprid exposure during the early-life larval phase perturbs honeybee gut microbiota by day 6, evident in the reduced ecological niche width compared with the first day across various treatment groups. This suggests a decrease in species diversity, likely due to increased competition. Initially, thiacloprid appears to amplify competition within the bee gut microbiota, as shown by the reduced niche width on day 1. However, by day 6, this competitive pressure seems to lessen, allowing the microbial community to stabilize, probably aided by the consistent sugar-water supply [52]. Meanwhile, we discovered that on day 1, thiacloprid alters the way the microbial community is structured, moving from a deterministic process to a stochastic process, which potentially affects the physiology and immunity of honeybees, supporting similar findings from our previous study [27]. However, by day 6, deterministic processes regain dominance, suggesting a return to stability in the gut flora.

This study was conducted under laboratory conditions, which had constraining factors and could not fully reflect the complex field conditions, such as multiple stressors and hive interactions. Consequently, the results may not accurately represent the real-world long-term exposure risks to honeybee health and gut microbiota [53]. Therefore, conducting field experiments in the future is crucial to investigating the long-term toxic effects of neonicotinoids on honeybees.

## 5. Conclusions

In summary, the present study examines the impact of sublethal thiacloprid larval exposure on emerged adult honeybee survival, sucrose intake, and gut microbiota composition in a laboratory setting. The data suggest that thiacloprid exposure modifies bee survival rates and sucrose intake, leading to alternations in intestinal microbial diversity, which are pronounced on the initial day post-emergence and tend to stabilize by the sixth day. Our research highlights the significance of assessing the long-term effects of thiacloprid application on bee health, emphasizing the necessity for implementing strategies that reduce negative consequences on bee health and ecosystem stability.

## Figures and Tables

**Table 1:** db-RDA analysis explained the relative influence of environmental factors on the composition of honeybee microbial communities in thiacloprid treatments.

	CAP1	CAP2	r^2^	*p*-Values
thiacloprid level	0.119	−0.993	0.699	0.001
age	0.989	0.150	0.967	0.001
thiacloprid	0.104	−0.995	0.300	0.001
weight	1.000	0.009	0.071	0.136

## Data Availability

All raw sequencing data have been deposited in the NCBI database (Bioproject ID: PRJNA940397).
